# Extreme Resistance to Viruses in Potato and Soybean

**DOI:** 10.3389/fpls.2021.658981

**Published:** 2021-04-06

**Authors:** Brian T. Ross, Nina K. Zidack, Michelle L. Flenniken

**Affiliations:** ^1^Department of Plant Sciences and Plant Pathology, Montana State University, Bozeman, MT, United States; ^2^Montana State Seed Potato Certification Lab, Department of Plant Sciences and Plant Pathology, Montana State University, Bozeman, MT, United States

**Keywords:** antiviral defense, extreme resistance, hypersensitive response, plant NLRs, plant viruses, potato, potato virus Y, soybean mosaic virus

## Abstract

Plant pathogens, including viruses, negatively impact global crop production. Plants have evolved complex immune responses to pathogens. These responses are often controlled by nucleotide-binding leucine-rich repeat proteins (NLRs), which recognize intracellular, pathogen-derived proteins. Genetic resistance to plant viruses is often phenotypically characterized by programmed cell death at or near the infection site; a reaction termed the hypersensitive response. Although visualization of the hypersensitive response is often used as a hallmark of resistance, the molecular mechanisms leading to the hypersensitive response and associated cell death vary. Plants with extreme resistance to viruses rarely exhibit symptoms and have little to no detectable virus replication or spread beyond the infection site. Both extreme resistance and the hypersensitive response can be activated by the same NLR genes. In many cases, genes that normally provide an extreme resistance phenotype can be stimulated to cause a hypersensitive response by experimentally increasing cellular levels of pathogen-derived elicitor protein(s). The molecular mechanisms of extreme resistance and its relationship to the hypersensitive response are largely uncharacterized. Studies on potato and soybean cultivars that are resistant to strains of Potato virus Y (PVY), Potato virus X (PVX), and Soybean mosaic virus (SMV) indicate that abscisic acid (ABA)-mediated signaling and NLR nuclear translocation are important for the extreme resistance response. Recent research also indicates that some of the same proteins are involved in both extreme resistance and the hypersensitive response. Herein, we review and synthesize published studies on extreme resistance in potato and soybean, and describe studies in additional species, including model plant species, to highlight future research avenues that may bridge the gaps in our knowledge of plant antiviral defense mechanisms.

## Plant Antiviral Defense

The majority of plants are sessile, and thus there are strong selective pressures on the accurate, rapid sensing and response to pathogen or parasite infection (Jones and Dangl, 2006). Understanding these immune processes are of paramount importance to humans, as plants are the foundation of both the Earth’s terrestrial ecosystems and the world economy. Of particular concern to humans are pathogens of crop species. Fungi, bacteria, and viruses are major threats and cause substantial crop losses. Controlling viral infection and spread in agricultural settings is challenging due to lack of chemical controls, rapid evolution of viruses, and transmission by insect vectors (Tian and Valkonen, 2013; Tromas et al., 2014). Genetic resistance (i.e., naturally evolved, breeder selected, and engineered) is a sustainable form of virus mitigation.

In plants, virus resistance is often controlled by dominantly inherited genes that encode nucleotide-binding leucine rich repeat proteins (NLRs; Kourelis and van der Hoorn, 2018). Most plant NLR proteins are composed of three primary domains: an N-terminal coiled-coil, Toll/Interleukin-1 receptor, or divergent coiled-coil domain; a central nucleotide-binding domain; and a C-terminal leucine-rich repeat (LRR) domain (Tamborski and Krasileva, 2020). These proteins function as intracellular immune receptors and their ability to elicit an immune response is controlled in part by nucleotide binding state. Specifically, inactive NLR proteins are bound to ADP, whereas recognition and binding of a pathogen effector protein allows for an NLR to transition to an active, ATP-bound state that is able to initiate an immune response (Takken et al., 2006; Wang et al., 2019a,b).

Nod-like receptors in animals share structural and functional similarities with plant NLRs but are a result of convergent evolution (Urbach and Ausubel, 2017). Animal Nod-like receptor proteins oligomerize in the cytoplasm after pathogen detection, triggering the formation of plasma membrane pores, release of inflammatory cytokines, and cell death (Shi et al., 2017). The formation of NLR homo- and heterodimer protein complexes are often required for immune activation and downstream signaling (Wang et al., 2019a,b). “Resistome” structures formed by Toll/Interleukin-1 receptor NLRs hydrolyze cellular nicotaminide adenine dinucleoside (NAD), initiating immune and cell death responses (Ma et al., 2020; Martin et al., 2020). Resistomes formed by coiled-coil NLRs resemble bacterial pore-forming structures. How coiled-coil resistomes induces immunity in plants remains unknown, but it is possible that it forms a membranous pore to induce cell death, similar to animal NLRs (Adachi et al., 2019a,b). Plant NLR-mediated immune responses mounted against numerous pathogens often result in localized cell death – a reaction termed the hypersensitive response (HR). Plant immune responses that result in cell death can range in appearance from localized, microscopic lesions (“micro-HR”) to the death of the entire plant (“systemic HR”; Balint-Kurti, 2019). The HR may exist as a point on a spectrum of plant physiological responses to pathogens (Bendahmane et al., 1999). A typical HR lies somewhere phenotypically between micro-HR and systemic HR, with cell death often occurring in the infection site and immediate vicinity.

Although commonly used as a sign of an active defense response, cell death is not necessarily a vital component of virus resistance mechanisms (Bendahmane et al., 1999; Komatsu et al., 2010). What has been often categorized as another type of resistance, termed “extreme resistance,” exists opposite systemic HR on the spectrum of plant antiviral immune responses. Extreme resistance is characterized by a lack of symptoms, and limited or lack of pathogen replication and pathogen spread (Bendahmane et al., 1999). Although the relationship between HR and extreme resistance is not well defined, in at least one example, the same NLR gene can initiate both extreme resistance and HR, depending on the amount of the pathogen elicitor protein recognized in the cell (Bendahmane et al., 1999). Extreme resistance and HR may be phenotypic variations of the same response pathway gradient or separate pathways that are sequentially activated. Plants can prime distal tissues for infection after pathogen recognition, resulting in a physiological state termed “systemic acquired resistance,” which serves to limit future infections beyond the infection site (Liu et al., 2010; Fu and Dong, 2013). Systemic acquired resistance can be initiated through both extreme resistance and HR, further indicating functional overlap between the two responses. Plant immune responses beyond the initial NLR-mediated pathogen recognition steps require further characterization. A mechanistic understanding of extreme resistance will further our understanding of plant immune responses to pathogens.

The incorporation of genes that control extreme resistance into favored crop cultivars is a focus of traditional and molecular breeding programs worldwide, particularly in crop species that are vulnerable to losses caused by virus infection. Research efforts have primarily focused on the identification and investigation of the genes and mechanisms that confer extreme resistance to viruses in potato (*Solanum tuberosum*) and soybean (*Glycine max*). There are examples of extreme resistance from other species as well, including *Arabidopsis thaliana*, an important model system. Notable genes conferring extreme resistance include Resistance to Potato virus X 1 (*Rx1*) and Resistance to Potato virus Y (PVY) from *Solanum stoloniferum (Ry_sto_*) genes in potato, which provide resistance to certain strains of Potato virus X (PVX) and PVY, and *Rsv* genes in soybean, which provide resistance to certain Soybean mosaic virus (SMV) strains. This review summarizes existing knowledge of the molecular mechanisms of plant-evolved extreme resistance to viruses.

## Extreme Resistance to Virus in Potato

### Research on *Rx1* in Potato Has Illuminated the Initial Molecular Steps of Extreme Resistance

The initial processes of extreme resistance are best-characterized in the potato *Rx1*-PVX system. Also referred to as *Rx*, *Rx1* is a coiled-coil NLR protein that confers extreme resistance to the *Potexvirus*, PVX, which can cause yield losses of up to 20% and is a common problem for potato growers (Adams et al., 1985). The *Rx1* gene is located on chromosome XII and has been introgressed from the wild species of potato, *Solanum andigena*, into some commercial potato cultivars (e.g., cv. Cara, Atlantic; Ritter et al., 1991; Bendahmane et al., 1997). The *Rx1* gene shares high sequence complementarity and is functionally redundant to another NLR, *Rx2*, which is found on potato chromosome V and was introgressed into potato from *Solanum acuale* (Ritter et al., 1991; Bendahmane et al., 1997). Despite similarities between the two genes, *Rx1* has received a much greater research focus than *Rx2*.

**Figure 1:**
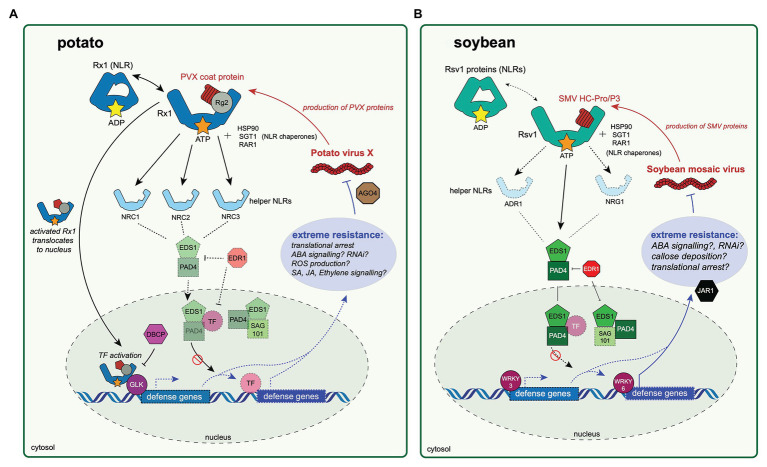
Extreme resistance to viruses in potato and soybean hosts. The potato antiviral extreme resistance response to Potato virus X (PVX) is conferred by Rx1 in potato **(A)** and extreme resistance to soybean mosaic virus (SMV) is conferred by genes within the *Rsv1* locus in soybean **(B)**. The first step in immune activation in either pathway relies on an nucleotide-binding leucine-rich repeat protein (NLR) protein, Rx1 in potato **(A)** or an Rsv1 protein in soybean **(B)**, directly or indirectly recognizing a pathogen-produced effector protein. The PVX coat protein is likely recognized after binding a RANGAP 2 (Rg2) protein “guarded” by Rx1, although this relationship is yet to be experimentally validated. After pathogen recognition, the activated (ATP-bound) Rx1 then translocates to the nucleus, at which point it binds a GOLDEN 2-LIKE transcription factor (GLK). The activity of Rx1-GLK complex is likely negatively regulated by a bromodomain-containing protein (DBCP). The Rx1-GLK complex binds DNA and may regulate expression of defense genes associated with the extreme resistance phenotype, although the genes that Rx1-GLK regulates are not known. The activated Rx1 protein relies on helper NLR proteins (NRC1, NRC2, and NRC3) to transmit an immune response. Rx1-mediated extreme resistance causes translational arrest of the PVX genome to occur, possibly through involvement of *ARGONAUTE 4*. **(B)** The SMV Helper-component protease (HC-Pro) or P3 protein is recognized directly or indirectly by an Rsv1 protein. Gene silencing of *ENHANCED DISEASE SUSCEPTIBLITY 1* (*EDS1*), *PHYTOALEXIN-DEFICIENT 4* (*PAD4*), *JASMONIC ACID-AMINO ACID SYNTHETASE* (*JAR1*), or *ENHANCED DISEASE RESISTANCE 1* (*EDR1*) abrogates the resistance response. Similarly, silencing of *WRKY3* and *WRKY6* disables resistance. In these diagrams, experimentally validated proteins involved in extreme resistance are highlighted, while proteins not validated but likely involved in extreme resistance responses are faded and have a dashed outline.

Previous research on NLR protein function indicates that most, if not all, NLRs rely on chaperone proteins for stability and proper function (Kadota et al., 2010). A triad of highly conserved NLR chaperone proteins, SUPRESSOR OF G2 ALLELE OF SKP1 (SGT1), HEAT SHOCK PROTEIN 90 (HSP90), and REQUIRED FOR MLA12 RESISTANCE (RAR1) are essential for disease resistance. Many studies involve the expression of *Rx1* in the genetically tractable model plant, *Nicotiana benthamiana* (*Nb*). Silencing of *NbSGT1* suppressed *Rx1*-mediated extreme resistance to PVX in *Nb*, and indicated that the extreme resistance conferred by Rx1 relies on proteins similar to non-extreme resistance conferring NLRs (Botër et al., 2007; Figure 1). Despite no known nuclear or cytoplasmic localization signals, Rx1 must be distributed in both the cytoplasm and nucleus for an extreme resistance immune response to occur (Slootweg et al., 2010; Tameling et al., 2010). The Rx1 coiled-coil domain is necessary for accumulation in the nucleus, while the LRR domain is required for cytoplasmic localization (Slootweg et al., 2018). Binding between Rx1 and *N. benthamiana* RAN GTPase ACTIVATING PROTEIN (*Nb*RG2) results in the retention of Rx1 in the cytoplasm, which is required for their dual roles in pathogen sensing and gene activation (Rairdan and Moffett, 2006; Sacco et al., 2007; Tameling et al., 2010).

The function of *Nb*RG2 in the *Nb*RG2-Rx1 complex is not clear, although a few possible roles are apparent (Slootweg et al., 2010; Hao et al., 2013). RanGAP proteins, like *Nb*RG2, facilitate the GTPase activity of Ran GTPase proteins, which aid in the transport of protein complexes into and out of the nucleus (Dasso, 2002). There are no known transport activities of RanGAP proteins themselves and *Nb*RG2 hydrolazing activity is not required for a successful resistance response (Tameling and Baulcombe, 2007). Many NLR proteins have evolved to monitor host proteins that are vulnerable targets of pathogen effector proteins (Collier and Moffett, 2009). Slootweg et al. (2010) suggest that *Nb*RG2 may act as a decoy target of the PVX coat protein, in which Rx1 would be “monitoring” *Nb*RG2 until bound by the PVX coat protein, after which Rx1 could initiate an immune response (Slootweg et al., 2010). This claim is buttressed by the findings that Gpa2, an NLR protein that shares a high amount of amino acid conservation with Rx1, guards *Nb*RG2 and initiates an immune response only when *Nb*RG2 is bound by effector proteins secreted by nematodes during infection (Sacco et al., 2009). Interactions between *Nb*RG2 and the PVX coat protein could produce a conformational change in *Nb*RG2, which could in turn allow Rx1 to reach an activated state (Hao et al., 2013). Many NLR proteins work in pairs with other NLRs or helper NLRs to effectively transmit immune signals (Adachi et al., 2019a). A study by Wu et al. (2017) found that silencing of three genes that encode helper NLR proteins, NLR REQUIRED FOR CELL DEATH 1 (NRC1), NRC2, NRC3, disabled *Rx1*-conferred extreme resistance, but only if all three genes were silenced simultaneously (Wu et al., 2017). These results indicate levels of redundancy and possible robustness to interference by pathogens within plant immune signaling.

Binding between Rx1 and PVX coat protein occurs in the cytoplasm (Bendahmane et al., 1995). Recognition of PVX coat protein by Rx1 and subsequent binding causes the release of *Nb*RG2 from Rx1, allowing Rx1 to translocate to the nucleus through a yet unknown process, as there are no detectable nuclear localization signals within Rx1 (Tameling and Baulcombe, 2007). However, nuclear localization signals are notoriously difficult to predict and can be hidden within secondary structure. Although many studies have focused on NLR functionality in the cytoplasm, there are other examples of NLR nuclear localization and function (Burch-Smith et al., 2007; Shen et al., 2007). The barley NLR, *MLA10*, interferes with repression of defense genes by binding to WRKY transcription factors in the nucleus (Chang et al., 2013). Experiments in which nuclear exclusion signals or nuclear localization signals were added to an Rx1-GFP fusion protein indicated that the DNA binding capabilities of Rx1 are contingent upon Rx1 recognition of the PVX coat protein in the cytoplasm, which is followed by movement of Rx1 to the nucleus. Resistance, but not cell death responses were compromised in experiments in which Rx1 was localized predominantly to the nucleus or the cytoplasm (Bendahmane et al., 1999; Knip et al., 2019; Richard et al., 2021). These results indicate that Rx1 must be activated (ATP-bound) in order to successfully bind DNA and likely must be able to translocate from the cytoplasm to the nucleus in order to initiate the extreme resistance response. Activation of Rx1 and other NLRs occurs after binding effector proteins, in this case the PVX coat protein. *In vitro* binding assays indicate that the DNA binding activity of Rx1 is inhibited, while in an inactivated state (ADP-bound), while the activated (ATP-bound) Rx1 can bind DNA (Fenyk et al., 2015). Further, recognition of the PVX coat protein likely results in a perturbed binding between the LRR and ARC2 (Apaf-1, R proteins, and CED-4) domains of Rx1, a process which may play a role in initiation of resistance pathways. Rairdan and Moffett (2006) suggest that the LRR domain can repeatedly dissociate and reassociate with the ARC2 domain after recognition of the PVX coat protein, and that this iterative process may serve to amplify the resistance signal and could play a key role in the extreme resistance response (Rairdan and Moffett, 2006).

Upon entering the nucleus, the activated nucleotide-binding domain of *Rx1* allows for binding and melting of double-stranded DNA in a non-sequence specific manner, but with a higher affinity for DNA topologies similar to transcription start site bubbles (Finzi and Dunlap, 2010; Tang et al., 2011; Townsend et al., 2018). The DNA binding activities of Rx1 likely become sequence-specific when in complex with *Nb*GLK1 (a Golden 2-like transcription factor), although this remains to be definitively proven (Townsend et al., 2018). The activation state of Rx1 likely determines the DNA binding activity of *Nb*GLK1, as inactivated Rx1 in complex with *Nb*GLK1 does not bind DNA *in planta* (Townsend et al., 2018). Golden 2-like transcription factors preferentially bind DNA sequences with GLK-like motifs and are known to regulate the transcription of genes involved in abscisic acid (ABA) signaling (Ahmad et al., 2019). Although the genes that *Nb*GLK1 regulates in response to PVX infection are not known, GLK-like transcription factors play a role in resistance to cucumber mosaic virus and fungal pathogens in *Arabidopsis* (Savitch et al., 2007; Murmu et al., 2014; Han et al., 2016).

There are likely other proteins that interact with Rx1 and *Nb*GLK1 in the nucleus. Sukarta et al. (2020) recently described a direct interaction between Rx1 and a DNA-binding bromodomain containing protein, *Nb*DBCP (Sukarta et al., 2020). The precise role(s) of *Nb*DBCP remains unclear, though it likely acts as a repressor of *Rx1*-mediated resistance signaling. Silencing of *Nb*DBCP as well as co-expression of non-functional *Nb*DBCP decreased PVX coat protein accumulation during Potato virus X infection in plants expressing Rx1, indicating that *Nb*DBCP may negatively regulate extreme resistance responses. Binding of DNA by *Nb*DBCP occurs *in situ*, but not when co-expressed with Rx1 or during PVX infection. Size exclusion chromatography results indicate that Rx1, *Nb*DBCP, and *Nb*GLK1 may form a transient complex; however, this idea remains theoretical and untested. These results conservatively indicate a negative regulatory role of *Nb*DBCP on *Rx1*-mediated extreme resistance, although its exact role(s) require more research (Sukarta et al., 2020).

Intriguingly, overexpression of *Nb*GLK1 in *N. benthamiana* confers immunity to PVX even in the absence of Rx1, and this immunity does not result in HR (Townsend et al., 2018). These results may signal that *Nb*GLK plays a role in controlling gene expression that is important for extreme resistance, but that role is likely independent or upstream of HR/cell death. Additionally, *Nb*DBCP overexpression, in the presence of Rx1 and during PVX infection, resulted in increased cell death. Expression of a non-functional *Nb*DBCP resulted in decreased cell death, lending credence to the idea that (1) *Nb*DBCP negatively regulates the extreme resistance pathway and (2) extreme resistance and HR/programmed cell death are largely separate or sequentially activated pathways (Sukarta et al., 2020). Similar separation of cell death and resistance has been reported in *N. benthamiana* plants expressing the barley NLR, *MLA10*, with a nuclear localization tag (Bai et al., 2012). The *MLA10* gene provides resistance to the barley powdery mildew fungus, indicating that nuclear functions of NLRs may not be specific to virus resistance.

Extreme resistance conferred by *Rx1* does not limit viral spread through the phloem. Grafting experiments revealed that PVX moved from a susceptible rootstock through the phloem of a middle, resistant scion and into another upper, susceptible scion and caused infection (Bendahmane et al., 1999). A reaction similar to *Rx1*-mediated extreme resistance can occur in protoplasts, while HR does not (Otsuki et al., 1972; Baulcombe et al., 1984; Goulden et al., 1993; Kohm et al., 1993; Bendahmane et al., 1995). These results may indicate that intercellular signaling components or cell wall components may not be necessary for *Rx1*-conferred extreme resistance responses, whereas they are for HR responses. It is likely that the mechanisms controlling extreme resistance occur rapidly in the cell, as extreme resistance prevents viral replication and spread beyond the initial point of inoculation.

Overexpression of Rx1 in *N. benthamiana* results in HR, regardless of whether its elicitor, the PVX coat protein, is present or not. Transformation of *Rx1* under its native promoter into *N. benthamiana* and *Nicotiana tabacum* results in a typical, symptomless extreme resistance to PVX, indicating that functionality and possible downstream interacting elements are conserved between species (Bendahmane et al., 1999). Overexpression of PVX coat protein in *N. benthamiana* expressing *Rx1* under its native promoter results in HR. Bendahmane et al. (1999) suggest that the continued production of the PVX coat protein after the initial recognition event and extreme resistance activation may signal to the cell that extreme resistance has been overcome and that further immune action may be warranted, hence the subsequent HR (Bendahmane et al., 1999). This hypothesis is supported by the finding that extreme resistance conferred by *Rx1* is epistatic to HR, as plants expressing both *Rx1* and *N*, an NLR that provides resistance with an HR phenotype to Tobacco mosaic virus, were resistant to Tobacco mosaic virus infection but did not display HR when the virus was engineered to express both the PVX coat protein and the protein elicitor of *N* during infection. The addition of a nuclear localization signal to *Nb*RG2 caused Rx1 to accumulate almost solely in the nucleus and prevented HR from occurring, even when auto-active *Rx1* mutants were overexpressed (Slootweg et al., 2010). Thus, it is likely that Rx1 must be located in the cytoplasm in order for HR and concurrent signaling to occur. This conclusion is congruent with cytosolic location of PVX replication and PVX coat protein detection by Rx1 (Slootweg et al., 2010).

The possible interconnectedness between HR and extreme resistance responses underscores the need for more sensitive resistance assays. To better understand extreme resistance, it is paramount that researchers try to replicate the native expression levels of *Rx1* (and other genes that confer extreme resistance) when experimenting outside of its native potato system. Slootweg et al. (2010) expressed *Rx1* from a vector with a second start codon inserted upstream and out of frame of the *Rx1* start codon. The resulting “leaky” expression of Rx1 led to protein levels in *N. benthamiana* that were 5–10x lower than expression driven by a typical CaMV35S promoter, and a much more sensitive assay (Slootweg et al., 2010). Studies in *A. thaliana* also indicate the expression level of NLR proteins may in part determine the phenotype of the resistance response. For example, resistance to the yellow strain of Cucumber mosaic virus is conferred by *RCY1*, a coiled-coil NLR, in *Arabidopsis* ecotype C24. Resistance to Cucumber mosaic virus (Y) *via RCY1* is normally accompanied by a hypersensitive response. Transgenic *Arabidopsis* lines that over-expressed *RCY1* at high levels (i.e., ~100x greater than its native promoter) exhibited the extreme resistance phenotype. Transgenic plants that expressed *RCY1* at moderately elevate levels (i.e., ~20x greater than native expression) exhibited enhanced resistance with very small areas of cell death (“micro-HR”; Sekine et al., 2008). The hypersensitive response was observed in transgenic lines with levels of *RCY1* expression similar to native expression. None of the *RCY1* transformed *Arabidopsis* lines became systemically infected with Cucumber mosaic virus (Y). These results indicate that *RCY1* expression levels, at least in part, govern virus-resistance phenotypes, possibly by determining the type of the subsequent immune response.

Other publications studying the NLR, HRT, which confers resistance to Turnip crinkle virus, have noted similar results (Cooley et al., 2000). However, levels of resistance protein expression are likely not the only factor governing immune responses. Overexpression of the Turnip crinkle virus coat protein, which is the binding target of HRT, resulted in severe HR, similar to the reaction that occurs after overexpression of the PVX coat protein plants expressing Rx1 (Cooley et al., 2000). Expression and activity levels of NLR proteins in plants are regulated in many ways (e.g., transcriptionally, post-transcriptionally, post-translationally, etc.; Borrelli et al., 2018). It is possible that increased expression of HRT or RCY1 is sufficient to overcome some negative regulation, resulting in faster immune responses.

The mechanistic details of Rx1 conferred resistance restricting PVX viral replication and spread are not yet known, however, experiments indicate that the translational arrest of the PVX transcripts is likely a major component of these resistance processes (Richard et al., 2021). By employing an inducible effector protein expression system and nuclear- and cytoplasm-localized Rx1 expression, Richard et al. (2021) demonstrate that Rx1-conferred extreme resistance likely relies on PVX transcript-specific translational arrest and that this response occurs within a few hours after infection (Meteignier et al., 2016; Richard et al., 2021). These data also demonstrate that nuclear- or cytoplasm-localized Rx1 expressed individually or together, results in HR or trailing necrosis (i.e., HR that trails viral spread throughout the plant) after 4 hours of the induction of PVX coat protein transcription, but does not induce extreme resistance. These results further support that upon recognition of the PVX coat protein in the cytoplasm, Rx1 must translocate to the nucleus in order to initiate the extreme resistance response. Translational arrest is a common host antiviral strategy (Machado et al., 2017). *Rx1*-expressing, PVX-infected potato protoplasts did not support replication of either Tobacco mosaic virus or Cauliflower mosaic virus, indicating that the Rx1-mediated antiviral response was a general antiviral response (Goulden et al., 1993; Bendahmane et al., 1995). Further, Rx1 may not be unique in this regard, as recognition of the resistance elicitor, Tobacco mosaic virus p50, by the tobacco NLR gene, *N*, can initiate an immune response that prevents translation of PVX transcripts in *N. benthamiana*, but only in the presence of RNA containing the PVX coat protein coding sequence (Bhattacharjee et al., 2009).

These results collectively suggest that a conserved characteristic of viral RNAs, possibly secondary structure, may be specifically targeted by NLR-mediated translational inhibition responses and that this mechanism may play a key role in the extreme resistance response. Although the factor(s) governing translational arrest are not known, it is interesting to note that virus-induced gene silencing of *ARGONAUTE 4* (*AGO4*) disabled symptomless resistance responses, and in turn allowed systemic PVX infection in *N. benthamiana* (Bhattacharjee et al., 2009). Similarly, in *N. benthamiana* plants in which the RNA interference (RNAi) suppressor proteins from Beet western yellows virus and Turnip crinkle virus, P0 and P38, were expressed, the antiviral response was also disabled. The Turnip crinkle virus P0 protein targets and induces degradation of Argonaute proteins (Baumberger et al., 2007; Bortolamiol et al., 2007). The expression of other suppressors of RNAi, including potyviral Helper-component protease (HC-Pro), did not prevent an antiviral response from occurring, although HC-Pro disables RNAi through sequestration of virus-derived small RNAs, not through the degradation of Argonaute (Mallory et al., 2002). Another RNA virus, Tobacco rattle virus (TRV), expresses a suppressor of RNAi silencing protein, 16k, which binds AGO4 and *ago4* mutant *N. benthamiana* plants are more susceptible to infection (Ma et al., 2015; Fernández-Calvino et al., 2016).

ARGONAUTE 4 is well known for its roles in transcriptional gene silencing and the RNA-directed DNA methylation pathway, as well as methylation-based antiviral defense against plant viruses with DNA genomes (Zilberman et al., 2003; Li et al., 2006; Raja et al., 2008, 2014; Gao et al., 2010; Greenberg et al., 2011; Dowen et al., 2012; Wierzbicki et al., 2012; Ye et al., 2012). These well-characterized roles of AGO4 all occur in the nucleus. Interestingly, cytoplasm-localized AGO4 is necessary for resistance to the potexvirus virus, *Plantago asiatica* mosaic virus, in *Arabidopsis*. This resistance does not involve other protein components of the RNA-directed DNA methylation pathway (e.g., *DICER-LIKE 3*, *RNA POLYMERASE IV*, and *RNA POLYMERASE 5*), indicating that AGO4 antiviral activity in this case is likely independent of its DNA methylation activity (Brosseau et al., 2016). The importance of AGO-4 non-methylation-based defense is not limited to antiviral responses, as silencing of AGO4 in *Arabidopsis* plants without functional RNA-directed DNA methylation pathways increased susceptibility to *Pseudomonas syringae* (Agorio and Vera, 2007).

Research on *Rx1*-conferred extreme resistance has illuminated the potential nuclear functions of Rx1 and laid a framework for future studies. In particular, gaining an understanding of the DNA sequences targeted by the Rx1-*Nb*GLK1 complex and the possible transcriptional changes that occur after recognition of the PVX coat protein will aid in the identification of other genes and mechanisms involved in *Rx1*-conferred extreme resistance. Future experiments employing RNA sequencing, chromatin-immunoprecipitation sequencing, and translatome analysis would increase understanding of the regulation of this defense system. Additional studies are required to determine the role(s) of bromodomain containing proteins, to identify the DNA sequences that are targeted by the Rx1-GLK complex, and if targeted gene(s) are responsible for the next stages of the *Rx1* conferred extreme resistance response. Further, studies that dissect the antiviral translational repression response and possible antiviral roles of *AGO4* would provide a greater understanding of NLR-mediated immunity.

### Genes Conferring Extreme Resistance to Potato Virus Y Rely on Conserved Proteins That Are Also Necessary for HR

The *Ry* genes in potato (e.g., *Ry_sto_*, *Ry_fsto_*, and *Ry_adg_*) provide resistance to particular strains of PVX and PVY. The *Ry_sto_* gene (Resistance to PVY from *S. stoloniferum*), which conveys resistance to a broad spectrum of strains of PVY and Potato virus A (PVA) in potato and tobacco, is the only one of the genes controlling extreme resistance that has been isolated from the *Ry* loci (Cockerham, 1970; Barker, 1996; Grech-Baran et al., 2020). Global potato production is reliant on pathogen-free seed tubers, which are vulnerable to generational buildup and spread of pathogens, particularly viruses. Various PVY strains (including PVY^NTN^ and PVY^N-Wi^) are the most economically harmful viral pathogens involved in potato production and genetic resistance to PVY is a major focus of breeding programs (Karasev and Gray, 2013). Wild potato varieties and landraces are sources of PVY-specific NLR resistance genes that can be introgressed into commercial potato cultivars. Loci conferring extreme resistance to PVY have been mapped in *Solanum chacoense* (*Ry_chc_*), *S. tuberosum* group Andigena (*Ry_adg_*), and *S. stoloniferum* (*Ry*_*st*o_; Herrera et al., 2018). Grech-Baran et al. (2020) employed resistance enrichment sequencing (RenSeq) to isolate the gene conferring *Ry_sto_*-mediated extreme resistance from the commercial potato cultivar, Alicja. Introgressed from *S. stoloniferum*, Ry_sto_ is a Toll-interleukin receptor (TIR) NLR protein, similar to other potato virus resistance genes (e.g., *N*, *Pvr4*, *Y-1*, etc.). The broad-spectrum resistance conferred by *Ry_sto_* lends it an attractive trait for breeders of Solanaceous plants. The *Ry_sto_* gene is present in various commercial potato cultivars, including American cultivars Payette Russet and Castle Russet and European cultivars Alicja, White Lady, and Pirola. The Ry_sto_ protein either directly or indirectly recognizes or binds the coat protein of PVY and PVA to elicit the extreme resistance response (Grech-Baran et al., 2020).

The *Ry_sto_* gene has been cloned and expressed in PVY-susceptible Solanaceous plants. Challenge of transgenic plants expressing *Ry_sto_* under its native promoter with PVY usually results in an extreme resistance response (i.e., no infection, no symptoms), but can cause veinal necrosis or HR in response to some isolates of PVY^O^ (Hinrichs-Berger et al., 1999). Co-expression of *Ry_sto_* and the PVY coat protein under control of a CaMV35S promoter results in HR in potato and *N. benthamiana*. Expression of *Ry_sto_* in tobacco and subsequent challenge with PVY produced some localized necrosis in inoculated leaves. Grech-Baran et al. (2020) suggest that establishment of either extreme resistance or HR depends on at least three variables: expression level of the resistance gene; abundance of the cognate effector protein; and the genetic background of the host. Extreme resistance conferred by *Ry_sto_* relies on at least two other genes for successful immune activation: the lipase-like *ENHANCED DISEASE SUSCEPTIBILTY* (*EDS1*) and the CC-NLR, *N REQUIREMENT GENE 1* (*NRG1*). These dependencies corroborate studies on other TIR-NLRs (Grech-Baran et al., 2020).

Similar to the Rx1-PVX system, the genes controlling *Ry_sto_*-mediated extreme resistance downstream of virus recognition are not known, although some results have hinted at the involvement of proteins that interact with plasmodesmata. β-1,3-glucanase proteins aid in plant virus infection, likely by hydrolyzing callose and increasing the size exclusion limit of plasmodesmata, thus allowing for cell to cell spread of the virus (Iglesias and Meins, 2000). Callose is a polysaccharide that influences the size exclusion limit of the plasmodesmata and also serves as a deposition site for defense compounds (Zavaliev et al., 2011). Overexpression experiments of β-1,3-glucanase (class III) proteins were carried out in potato cultivars Santé (which displays extreme resistance to PVY) and Désirée (PVY-susceptible) within the context of PVY^NTN^ infection. Resistant Santé plants overexpressing *β-1,3-glucanase* exhibited modest, transient increases in PVY^NTN^ that dissipated within days of infection and the virus did not spread beyond the inoculated leaf. Susceptible Désirée plants that overexpressed *β-1,3-glucanase* may have exhibited slightly faster systemic infection of PVY^NTN^, although a relatively small sample size precluded more definitive conclusions (Dobnik et al., 2013).

Callose deposition is also targeted by PVY during infection, as PVY-encoded HC-Pro suppresses callose deposition during PVY^O^ infection through an unknown mechanism (Chowdhury et al., 2020). Callose deposition also occurs in cells surrounding HR activity during PVY infection and those cells can harbor viable PVY, thus further indicating that cell death is not the primary driver of resistance during HR and that callose deposition alone is not effective at arresting viral spread (Lukan et al., 2018). Cells undergoing HR/cell death processes may release signals to surrounding cells to initiate immune responses or that may act as defense compounds themselves (Lamb and Dixon, 1997). These signals may include reactive oxygen species (ROS), which are a common component of plant immune responses (Qi et al., 2017). Whether or not reactive oxygen signaling acts a component of extreme resistance is not known, although there are examples of symptomless resistance to Tobacco mosaic virus in tobacco plants induced by application of ROS (Kuenstler et al., 2016). The timing of foliar treatments was key to inducing resistance to Tobacco mosaic virus, as resistance did not occur in plants that were treated with reactive oxygen species 3 days after virus inoculation, but an HR-like reaction and cell death did.

The NLR resistance gene, *Ny-1*, also provides resistance to PVY, but the immune response is accompanied by HR. Extreme resistance conferred by *Ry_sto_* is epistatic to *Ny-1*-mediated HR, as plants expressing both *Ny-1* and *Ry_sto_* exhibit an extreme resistance phenotype but lack HR when challenged with a PVY^NTN^ isolate that is recognized by both Ry_sto_ and Ny-1 (Grech-Baran et al., 2020). For many TIR-NLR proteins, including *Ny-1*, resistance breaks down at high or low temperatures, while Ry_sto_ function is not limited by high ambient temperatures. Modulation of defense responses by temperature is likely controlled by NLR proteins, as point mutations in *NLR* genes can decrease nuclear accumulation of NLR proteins at higher temperatures and reduce NLR immune function (Zhu et al., 2010). At higher temperatures plants may preferentially activate pattern-triggered immunity (PTI), rather than NLR-dependent immunity (Cheng et al., 2013). Since NLR proteins confer resistance to plant pathogens that disable host pattern-triggered immune responses, a greater understanding of NLR thermosensitivity is needed as global temperatures continue to rise (Trebicki, 2020).

The expression levels of both the genes conferring extreme resistance and the viral elicitor protein seems to be a critical factor if HR or extreme resistance occurs in both Rx1-PVX and *Ry_sto_*-PVY systems. The cellular distribution of Ry_sto_ both before and during resistance responses is not known but should be the focus of further research given the potential nuclear functions of Rx1. The advent and expanded use of RenSeq, which allows for the expedited identification of NLR genes conferring specific resistance phenotypes, and CRISPR technologies, which allow for precise genome editing, should facilitate faster identification and breeding of resistance genes (Witek et al., 2016; Zhang et al., 2019). As extreme resistance is largely characterized by a lack of infection symptoms, there may be a pool of genes that confer extreme resistance to viruses that are yet to be discovered within landraces or wild Solanaceous species. For example, the *PVR4* gene, which encodes an NLR protein that originated in a landrace of hot pepper (*Capsicum annum*), confers extreme resistance to multiple potyviruses, including many PVY strains, Pepper mottle virus, and Pepper severe mosaic virus (Kim et al., 2015). The PVR4 protein recognizes the potyviral RNA-dependent RNA polymerase (NIb) to elicit the extreme resistance response (Kim et al., 2015). Global potato production relies on labor-intensive seed tuber certification programs to prevent pathogen accumulation, particularly viruses, with a large focus on PVY strains. Given that many NLR genes can be shuttled between *Solanaceous* species without a loss of function, transferring multiple, broad-spectrum NLR genes that target different potyviral components (i.e., *Ry_sto_*, *PVR4*) to potato could provide durable and sustainable immunity to potyviruses (Rivero et al., 2012; Zhu et al., 2012; Jo et al., 2014).

### Tobacco Rattle Virus-Caused Corky Ringspot Disease in Potato Is Likely the Result of an HR-Like Immune Response

Potyviruses are not the only problematic viruses of potato production. TRV, of the genus Tobravirus, causes necrosis in potato tubers, a disease commonly referred to as corky ringspot disease or “spraing.” Corky ringspot disease can render tubers unmarketable. Tobacco rattle virus is vectored by various species of nematodes and can infect many different plant species. Although cultivars that display symptoms of TRV infection in tubers are often labeled as susceptible, the ringed, necrotic tuber tissue characteristic of corky ringspot disease are likely the result of HR in response to viral infection in the tuber (Xenophontos et al., 1998; Sahi et al., 2016). Some frequently grown cultivars are tolerant to TRV infection and can contain high levels of virus throughout the plant and yet remain largely asymptomatic, although these tolerant infections can have negative effects on yield and tuber size and also serve as inoculum sources (Dale et al., 2000; Brown et al., 2009). The potato cultivar, Saturna, displays an extreme resistance response in leaves when inoculated with the TRV isolate, PpK20, and no symptoms or detection of TRV in tubers. A yet undescribed protein in Saturna recognizes the TRV movement protein, 29K, to initiate an extreme resistance response (Ghazala and Varrelmann, 2007). Similar to potato cultivars that display extreme resistance to PVY and PVX, overexpression of the elicitor protein (29k, in this case) leads to HR in the infected leaf.

Corky ringspot disease is a significant problem for growers in Europe, while TRV is a small but growing problem in the United States. Once TRV/corky ringspot disease occurs in a field it can be difficult to eliminate completely. Soil fumigation can reduce the presence of stubby root nematode vectors, but often does not completely eliminate TRV presence. Stubby root nematodes can feed on a wide variety of plants (i.e., potato, barley, corn, peas, brassicas, various common weeds, and others), often rendering crop rotations in infested fields non-effective at lowering vector pressure. Given that corky ringspot disease/spraing is caused by an HR-like immune response in tubers, then the largely symptomless immune response provided by extreme resistance would be a valuable asset to growers. Further, isolation of the gene(s) that confer extreme resistance to TRV in potato would be beneficial for breeding resistant cultivars.

## Extreme Resistance to Viruses in Soybean

The majority of the extreme resistance literature examines responses and pathways occurring after virus recognition in the soybean-Soybean mosaic virus pathosystem. SMV is a potyvirus that predominantly infects plants in the family *Fabaceae* (Hajimorad et al., 2018). SMV is classified into seven strains, denoted G1-G7, with virulence in soybean generally increasing with strain number (e.g., isolates of strains G5-G7 are generally the most virulent in soybean cultivars; Widyasari et al., 2020). Four dominant resistance loci, termed “Rsv,” (*Rsv1*, *Rsv3*, *Rsv4*, and *Rsv5*) are effective against various strains and isolates of SMV and are located on soybean chromosomes 2, 13, and 14. The genes conferring resistance from some of these loci have been identified. China uses a separate system to designate SMV strains and resistance genes, although many of the dominant resistance genes that have been identified by research teams in China (termed *Rsc* resistance genes) map to the same chromosomes and similar loci as Rsv resistance genes, their relationships remain largely uncharacterized (Wang et al., 2017; Figure 1).

The *Rsv1* and *Rsv5* loci are located within a complex region on the distal end of soybean chromosome 13 (Hayes et al., 2004). As *Rsv1* and *Rsv5* are tightly linked, they were once considered to be alleles of the same gene; however, they are likely two separate NLR genes (Klepadlo et al., 2017). Because *Rsv1* and *Rsv5* are often inherited together, it is possible that some interpretations of *Rsv1*-mediated resistance are complicated by involvement of an undetected *Rsv5* allele (Klepadlo et al., 2017). Extreme resistance conferred by *Rsv5* prevents infection by SMV-G1 (Klepadlo et al., 2017). The mechanism of *Rsv5*-mediated resistance is unknown and the gene responsible has not been isolated or cloned. Resistance provided by *Rsv4* is unique in that it is not conferred *via* an NLR-type protein, as *Rsv4* encodes an RNAse-H family protein with the ability to degrade dsRNA. Interactions between the SMV P3 protein and Rsv4 promote dsRNA degradation and prevent viral replication (Hayes et al., 2000; Maroof et al., 2010; Ishibashi et al., 2019). The ability of *Rsv4* to prevent infection declines with age, as mature plants are more susceptible to infection (Hayes et al., 2000; Maroof et al., 2010). This unique form of resistance appears to be independent of extreme resistance and HR, although it is phenotypically similar to extreme resistance.

The resistance conferred by *Rsv3* to SMV may be mechanistically different from *Rsv1*-conferred extreme resistance (Zhang et al., 2009). Specifically, *Rsv3*-expressing soybean plants inoculated with SMV-G7 expressing β-glucuronidase (GUS) exhibited small, isolated GUS expression foci at 5 days post inoculation, while *Rsv1*-plants inoculated with SMV-N expressing GUS did not (Zhang et al., 2009). *Rsv1* and *Rsv3*-expressing plants did not become systemically infected or exhibit cellular death; they were phenotypically indistinguishable from the mock-inoculated plants. Based on these results, Zhang et al. (2019) suggest that *Rsv1* and *Rsv3* provide resistance through functionally distinct immune responses. However, many publications describe *Rsv3*-conferred resistance as a form of extreme resistance despite the report by Zhang et al. (2009) (Seo et al., 2009, 2014, Khatabi et al., 2012; Ilut et al., 2016; Alazem et al., 2018). For consistency, herein, *Rsv3*-conferred resistance is referred to as a form of extreme resistance, recognizing that future studies may describe mechanistic differences between *Rsv3*-conferred and *Rsv1*-conferred resistance, as well as identify additional examples of NLR-conferred extreme resistance. Further experiments, including qPCR validation of limited SMV replication in *Rsv3*-expressing plants, as well as an extended time course comparison between *Rsv1*- and *Rsv3*-expressing plants directly after inoculation with various SMV strains expressing a reporter gene would likely produce more definitive conclusions.

### Virus-Induced Gene Silencing Experiments Have Illuminated Proteins Involved in Extreme Resistance Conferred by the *Rsv1* Locus

The *Rsv1* locus encodes multiple NLR genes, is highly complex, and is mapped to soybean chromosome 13 (Widyasari et al., 2020). The extreme resistance phenotype conferred by the *Rsv1* locus is contingent upon the strain of the infecting virus and the *Rsv1* allele present. There are 10 identified alleles of the *Rsv1* locus, which are associated with strain-specific resistance to SMV (Klepadlo et al., 2017; Wu et al., 2017). Various resistance phenotypes occur in plants containing the *Rsv1* locus that are infected with SMV strains G1–G7. The dominant *Rsv1* allele, which shares its name with the locus itself, confers extreme resistance to SMV strains G1–G6, but does not provide resistance to isolates of strain SMV-G7 and experimentally evolved SMV-G7d (Hajimorad et al., 2006).

Understanding of *Rsv1*-SMV interactions is limited because the gene(s) controlling *Rsv1*-conferred extreme resistance have not yet been identified. Gene silencing experiments concurrently targeting the expression of three non-Toll interleukin receptor NLR genes from the *Rsv1* allele of the *Rsv1* locus resulted in viral foci formation in resistant plants (cultivar L78–379), similar to that seen in susceptible plants (a near isogenic line of L78–379; Zhang et al., 2012). It is likely that at least two of those three targeted genes are necessary for *Rsv1*-conferred extreme resistance (Wen et al., 2013). Individual silencing of each gene was not possible because of high sequence similarity between the three (Zhang et al., 2012). Extreme resistance conferred by the *Rsv1* locus may be dependent on host recognition of two viral proteins, as mutations in both the HC-Pro and Protein 3 (P3) protein coding regions of the SMV genome are needed to break *Rsv1*-conferred extreme resistance (Hajimorad et al., 2005, 2006, 2008; Zhang et al., 2009; Wen et al., 2013).

Studies that utilized recombinant inbred soybean lines derived from the *Rsv1*-allele containing PI969893 line have enhanced understanding of *Rsv1*-conferred extreme resistance. One recombinant inbred line, L800, contains one NLR gene from the *Rsv1* locus, denoted as *3gG2*. Another recombinant inbred line, L943, contains five NLRs from the *Rsv1* locus, but does not contain *3gG2*. Interestingly, both lines are resistant to SMV-N, but L943 recognizes the HC-Pro from SMV-N to induce resistance, while L800 recognizes P3 from SMV-N to induce resistance, suggesting that the *Rsv1* locus may contain at least two resistance genes that recognize separate SMV proteins to induce resistance (Wen et al., 2013). It is interesting that line L943, which contains five NLRs from the *Rsv1* locus, allowed limited virus replication, as a few viral foci were evident by GUS staining after inoculation with SMV-N expressing GUS, but virus did not spread beyond the inoculated leaf and the foci did not grow. In contrast, line L800, which only contains one NLR (3gG2) displayed no GUS expression when inoculated with SMV-N expressing GUS (Wen et al., 2013). These small GUS foci are similar to those seen when comparing *Rsv1* and *Rsv3* extreme resistance in Zabala et al. (2009) and Zhang et al. (2009). These results could indicate that separate mechanisms may inhibit viral replication and viral spread and that both may be induced by the extreme resistance response.

Mutations in the SMV-N HC-Pro and P3 coding regions, which are recognized by Rsv1 proteins, enabled virus replication in L943 and L800 soybean lines, but not in P196983, which contains the entire *Rsv1* locus. An additional mutation to SMV-N HC-Pro resulted in productive infections in L800, L943, and PI96983 soybean lines, further indicating that the *Rsv1* locus likely contains multiple NLR or other genes that induce extreme resistance SMV. This idea is also supported by data indicating that SMV-N more easily evolves to evade variants that infect the single NLR (i.e., 3gG2) containing L800 soybean line than the L943 soybean line that contains five NLRs from *Rsv1* or the full *Rsv1* locus-containing PI96983 line (Hajimorad et al., 2003; Wen et al., 2013).

The HC-Pro and P3 genes are next to each other in the potyviral genome and on the resulting polyprotein before self-cleavage. It is not known if the protein(s) from the *Rsv1* locus recognize the SMV polyprotein or mature HC-Pro or P3 (Hajimorad et al., 2008; Wen et al., 2013). In another example of extreme resistance outside of potato or soybean, the cowpea cultivar, Arlington, displays extreme resistance to Cowpea mosaic virus by recognizing the enzymatically active 24K-protease as it cleaves the polyproteins of the Cowpea mosaic virus’ segmented genomes (Fan et al., 2011). It is also possible that closely related NLR genes from the *Rsv1* locus guard host proteins that are targeted by HC-Pro/P3 during the early stages of SMV infection (Hajimorad et al., 2008). It is also plausible that *Rsv1* contains multiple genes that confer variable levels of SMV resistance. For example, extreme resistance in the *3gG2* containing L800 soybean line could involve SMV-N P3 recognition, while other genes within the *Rsv1* locus could confer less effective resistance phenotypes (i.e., limited viral replication, but no spread) through SMV-N HC-Pro recognition. Further, line PI96983 could contain a yet unidentified NLR that recognizes a separate region of HC-Pro to induce extreme resistance.

Additional studies provide some indirect support that NLR proteins are likely involved in *Rsv1*-conferred extreme resistance to SMV, as silencing of genes that interact with NLR proteins resulted in increased virus load in resistant plants. Heat shock proteins often serve as molecular chaperones and HEAT SHOCK PROTEIN 90 (HSP90) acts as a chaperone to NLR proteins in plants and animals (Kadota et al., 2010). Virus induced gene silencing (VIGS) of *HSP90* resulted in SMV infection foci in the leaves of resistant cultivars, resulting in a phenotype similar to SMV infection in a susceptible cultivar (Zhang et al., 2012). Two other genes, *RAR1* and *SGT1*, serve as co-chaperones to HSP90 to stabilize NLR proteins. Silencing of *RAR1* and *SGT1* in two independent publications provided conflicting results as to if either are involved in extreme resistance (Fu et al., 2009; Zhang et al., 2012). These differences are likely explained by both differences in experimental design and in that gene silencing assays rarely result in a complete loss of target gene expression.

Experimental VIGS was also used to target a suite of other defense-related genes, including soybean homologs of *EDS1*, *PHYTOALEXIN DEFICIENT 4* (*PAD4*), *ENHANCED DISEASE RESISTANCE 1* (*EDR1*), and *JASMONIC ACID-AMINO SYNTHETASE 1* (*JAR1*). Reducing expression of the aforementioned genes resulted in SMV infection foci in the inoculated leaves of extreme resistant soybean cultivar L78–379 (Zhang et al., 2012). These infections were phenotypically similar to infections in SMV-infected leaves of susceptible cultivars, thus indicating that the silenced genes are likely components of the extreme resistance defense response. The *EDS1* protein family includes *EDS1*, *PAD4*, and *SENESCENCE-ASSOCIATED GENE 101* (*SAG101*). Heterodimers between EDS1/PAD4 and EDS1/PAD4/SAG101 are required for effector triggered immunity (ETI) in most seed plants. These heterodimers act downstream of pathogen recognition but upstream of transcription of defense genes. Recent research indicates that EDS1/PAD4 promote salicylic acid (SA) biosynthesis *via* the isochorismate pathway, but also control and preserve SA signaling through an alternative, parallel pathway. A third, separate salicylic acid signaling pathway relies on MAPK signaling (Cui et al., 2017). Salicylic acid is a primary signaling component of pattern-triggered and effector-triggered plant immune responses to many biotrophic pathogens and is therefore a primary target of plant pathogen interference. Downstream signaling and transcriptional reprogramming during ETI is controlled in part by the EDS1/PAD4 complex, which is in turn negatively regulated by the MAPKK kinase, *EDR1* (Neubauer et al., 2020). Salicylic acid-mediated disease resistance is negatively regulated by *EDR1* and *Arabidopsis EDR1* mutants are sensitive to ABA. These results are surprising because reducing expression of *EDR1* would not be expected to affect the resistance phenotype. Jasmonic acid (JA) signaling, which modulates defense responses to herbivory and often acts antagonistically to the salicylic acid pathway, is controlled in part by *JAR1* (Suza and Staswick, 2008).

Other genes targeted by VIGS within the context of *Rsv1*-conferred extreme resistance to SMV infection of soybean plants include WRKY transcription factors. WRKY proteins are among the largest transcription factor families in plants and largely regulate gene expression in response to abiotic and biotic stressors. A large scale VIGS study targeting 62 separate WRKY transcription factors revealed two genes, *GmWRKY30* and *GmWRKY6*, that compromised *Rsv1*-mediated resistance in soybeans that had been challenged with SMV (Zhang et al., 2012). *Arabidopsis WRKY6* is induced upon infection with a variety of viruses, which may suggest a conserved role for *WRKY6* within the context of virus infection across plant species (Whitham et al., 2003). *GmWRKY30* shares sequence similarity with *Arabidopsis WRKY3*, which is induced by pathogen infection and salicylic acid treatment in *Arabidopsis* (Liu et al., 2004; Lai et al., 2008; Pandey and Somssich, 2009). Further research is needed to understand which genes are regulated by *GmWRKY30* and *GmWRKY6* in response to viral infection and if similar antiviral roles are played by WRKY homologs in other plant species.

The factors controlling the relationship(s) between resistance, SMV strain, and *Rsv1* locus are not well understood and raise some interesting questions. Namely, why do some *Rsv1* alleles provide extreme resistance to particular SMV strains and others do not? For example, the *Rsv1* allele, *Rsv1-m*, provides extreme resistance to SMV strains G1, G4, and G5, but exhibits systemic necrosis when infected with G2, G3, G6, and G7 (Chen et al., 1991). The *Rsv1* allele confers resistance to SMV strains G1–G6, but systemic necrosis occurs in response to SMV-G7 infection. These differences may be attributed to allelic differences in elicitor binding/recognition efficiencies but may also be due to non-NLR host factors (Yuan et al., 2020). The cause(s) of systemic necrosis is not well understood but may be due to the delayed activation of the immune response/HR (Hajimorad and Hill, 2001; Hajimorad et al., 2003; Seo et al., 2009). In studies by Chen et al. (2017) and Wu et al. (2017), *Rsv-*1-containing soybean plants in which *EUKARYOTIC TRANSLATION INITIATION FACTOR 5A* was silenced exhibited increased virus accumulation and less necrosis, indicating a possible role for *EUKARYOTIC TRANSLATION INITIATION FACTOR 5A* in defense responses.

These plants also exhibited lower expression of other defense genes and genes involved in ROS signaling. Genetic variation in the RNA interference machinery between species and cultivars can result in differential susceptibility to virus infection. For example, the *ARGONAUTE 2* (*AGO2*) gene in *A. thaliana* limits PVX infection, but *N. benthamiana AGO2* does not. Further, *AGO2* exhibits a high incidence of polymorphism between *A. thaliana* accessions, some of which affect antiviral activity. The *AGO2* sequences contain signatures of selective pressure, possibly due to co-evolution with viruses (Brosseau et al., 2020). Variation among non-NLR host factors may explain some of the differences observed between resistance phenotypes of different species or cultivars (i.e., plants that develop viral foci in inoculated leaves but not systemic infection) but more research is needed to understand the impact of these differences on antiviral defense in plants.

Future studies involving the cloning and testing of individual genes within the *Rsv1* locus and identification of possible plant protein binding partners and other host factors involved in antiviral defense could further our understanding of Rsv1-conferred extreme resistance. Isolation of genes within the *Rsv1* locus could also provide access to a suite of genes that confer varying levels of resistance, which may be experimentally valuable.

### *Rsv3*-Mediated Extreme Resistance Response in Soybean Highlights Importance of Abscisic Acid Pathway

The gene *GLYMA.14g204700* (referred to hereafter as simply “*Rsv3*”), which encodes a coiled-coil NLR, is likely the gene responsible for *Rsv3*-mediated extreme resistance in the L29 cultivar (Tran et al., 2018). The soybean cultivar, L29, exhibits extreme resistance to isolates of SMV strains G5, G6, G7, and G5H *via* the *Rsv3* locus but is susceptible to the SMV-G7H isolate. The cylindrical inclusion protein of SMV is indirectly recognized by Rsv3 to initiate an immune response. Virus strain SMV-G7H escapes *Rsv3*-mediated host detection *via* amino acid substitutions in the cylindrical inclusion protein (Seo et al., 2009).

Transcriptomic responses of the L29 soybean cultivar to SMV strains have been the focus of recent studies investigating the mechanism of extreme resistance to SMV (Seo et al., 2014; Alazem et al., 2018, 2019). Alazem et al. (2018) postulate that the extreme resistance response may inhibit SMV replication and spread in three successive steps: (1) virus recognition by an NLR, resulting in rapid callose deposition at the plasmodesma of infected cells; (2) dsRNA detection, which induces viral genome destruction *via* RNAi; and (3) clearance of remaining viral proteins *via* the transient induction of autophagocytosis. Seo et al. (2014) compared the transcriptomic responses of L29 soybean plants when infected with either a virulent SMV strain (SMV-G7H) or an avirulent strain (SMV-G5H) at 8, 24, and 54 hours post infection (hpi). Analysis of differential gene expression revealed that genes encoding Type 2C protein phosphatases were among the most differentially expressed in L29 soybean plants that exhibited extreme resistance to avirulent SMV infection. Type 2C protein phosphatases are a large class of serine/threonine phosphatases that are key regulators of the ABA signaling network in plants (Fuchs et al., 2013). Abscisic acid signaling plays key roles in developmental regulation, stress responses, and likely defense responses (Flors et al., 2005; Fan et al., 2009; Alazem et al., 2017; Alazem and Lin, 2017; Xie et al., 2018; Pasin et al., 2020). L29 plants that overexpressed *PROTEIN PHOSPHATASE2C 3A* (*GmPP2C3a*) were not systemically infected when inoculated with virulent SMV, whereas all non-transgenic plants were systemically infected. Further analyses indicated that Type 2C protein phosphatases are likely regulators of the *Rsv3*-mediated extreme resistance response (Seo et al., 2014).

The aforementioned transcriptomic data was further analyzed in Alazem et al. (2018). Genes involved in the ABA pathway exhibited increased expression at 8 and 24 hpi in plants infected with avirulent SMV, but expression of these genes was not increased by virulent SMV infection. The induction of genes involved in ABA signaling in plants infected with avirulent SMV dissipated by 54 hpi. Topical application of ABA to L29 soybean leaves 24 h before infection with virulent SMV reduced virus accumulation by about 50% compared to non-treated plants, although virus replication was not completely eliminated. The specific mechanism(s) of ABA mediated virus reduction are an active area of research and are not well understood. It is likely that ABA signaling works in concert with other defense pathways including RNA interference and PTI. Foliar ABA application of *N. benthamiana* plants induced the expression of genes involved in RNAi in response to Bamboo mosaic virus infection, specifically the argonaute protein encoding genes, *AGO2* and *AGO3* (Alazem et al., 2017). There are similar reports of increased expression of RNAi pathway components in *Arabidopsis* and soybean (Chen et al., 2013; Alazem et al., 2014; Alazem and Lin, 2017).

One effect of greater cellular ABA concentrations is increased callose deposition at plasmodesmata (Nishimura et al., 2003; Flors et al., 2005; Li et al., 2012). Abscisic acid negatively regulates *β-1-3-glucanase* expression, which encodes for proteins that break down callose tissue. The number of *β-1-3-glucanase* transcripts was reduced at all three time points in plants infected with avirulent SMV. Increased callose deposition at inoculation sites occurred in L29 plants infected with avirulent SMV and in L29 plants overexpressing *GmPP2C3a* and infected with virulent SMV. Similar increases in callose deposition were not observed in non-transgenic plants infected with virulent SMV. Foliar treatments of L29 leaves with the callose synthesis inhibitor, 2-deoxy-D-Glucose (DDG), resulted in increased avirulent SMV abundance in treated leaves. These experiments elegantly illustrate the importance and interplay of ABA signaling, *GmPP2C3a* expression, and callose deposition to the extreme resistance response as conferred by *Rsv3* in soybean.

Cellular concentrations of ABA increased dramatically in L29 plants that exhibited extreme resistance, but the abundance of another plant defense signaling molecule, SA, did not change (Seo et al., 2014). The importance of SA signaling to antiviral defense in plants is well documented (Vleesschauwer et al., 2014). Increased SA accumulation occurred in L29 plants infected with virulent SMV but remained unchanged in plants infected with avirulent SMV, possibly suggesting that the antiviral role(s) of SA are not activated until after extreme resistance mechanisms have been broken. The ABA and SA pathways seem to interact in dueling antagonistic manner (i.e., high cellular ABA concentrations reduce SA biosynthesis and high SA concentrations inhibit ABA signaling; Nishimura et al., 2003; Zabala et al., 2009; Manohar et al., 2017). Interestingly, type 2C protein phosphatases (PP2Cs) bind both ABA and SA and may be important in modulating ABA and SA signaling (Manohar et al., 2017). ABA signaling is negatively regulated by Type 2C protein phosphatases which, in the absence of ABA, dephosphorylate ABA signaling kinases. Increased ABA concentration enhances binding between PP2Cs and PYR1-like regulatory elements, which inhibit PP2C dephosphorylation activity, resulting in the autophosphorylation of ABA signaling kinases and the expression of ABA responsive genes (Manohar et al., 2017).

Many SA-binding proteins have been documented. It is likely that a multitude of proteins are directly involved in regulating ABA and SA signaling. Staining leaves with DAB (3,3'-diaminobenzidine), which indicates reactive oxygen species presence, revealed that the *Rsv3*-mediated extreme resistance response does not rely on reactive oxygen species production. This result is contradictory to evidence indicating that reactive oxygen production is an important component of other plant immune responses, including extreme resistance. Therefore, it supports the idea that *Rsv3*-mediated extreme resistance differs mechanistically from *Rsv1*-mediated extreme resistance (Zhang et al., 2009). JA is also an important defense signaling molecule during plant-pathogen interactions, particularly during interactions with herbivorous insects (Suza and Staswick, 2008). The relative importance of JA signaling during viral infection remains unclear. Genes involved in the JA pathway exhibited either no change or decreases in expression in reactions with avirulent SMV but saw increased expression during virulent SMV infection at all timepoints. These data suggest that the induction of JA signaling may be important for establishing infection or may act as another layer of defense signaling. Another recent study by Alazem et al. (2019) illustrated that the effects of ABA treatment can be strain-dependent in cultivars lacking the *Rsv3* locus. Topical treatment of leaves with ABA reduced the severity of virulent SMV infection but promoted avirulent SMV infection in an *Rsv3*-lacking soybean cultivar. The presence of *Rsv3* (along with other proteins involved in its network, potentially) is necessary to fully recapitulate the extreme resistance response. It is plausible that avirulent SMV (strain G5) has evolved to manipulate components of the ABA pathway for its own benefit, but more research is needed to understand these differences (Alazem et al., 2019).

## Summary

**Figure 2:**
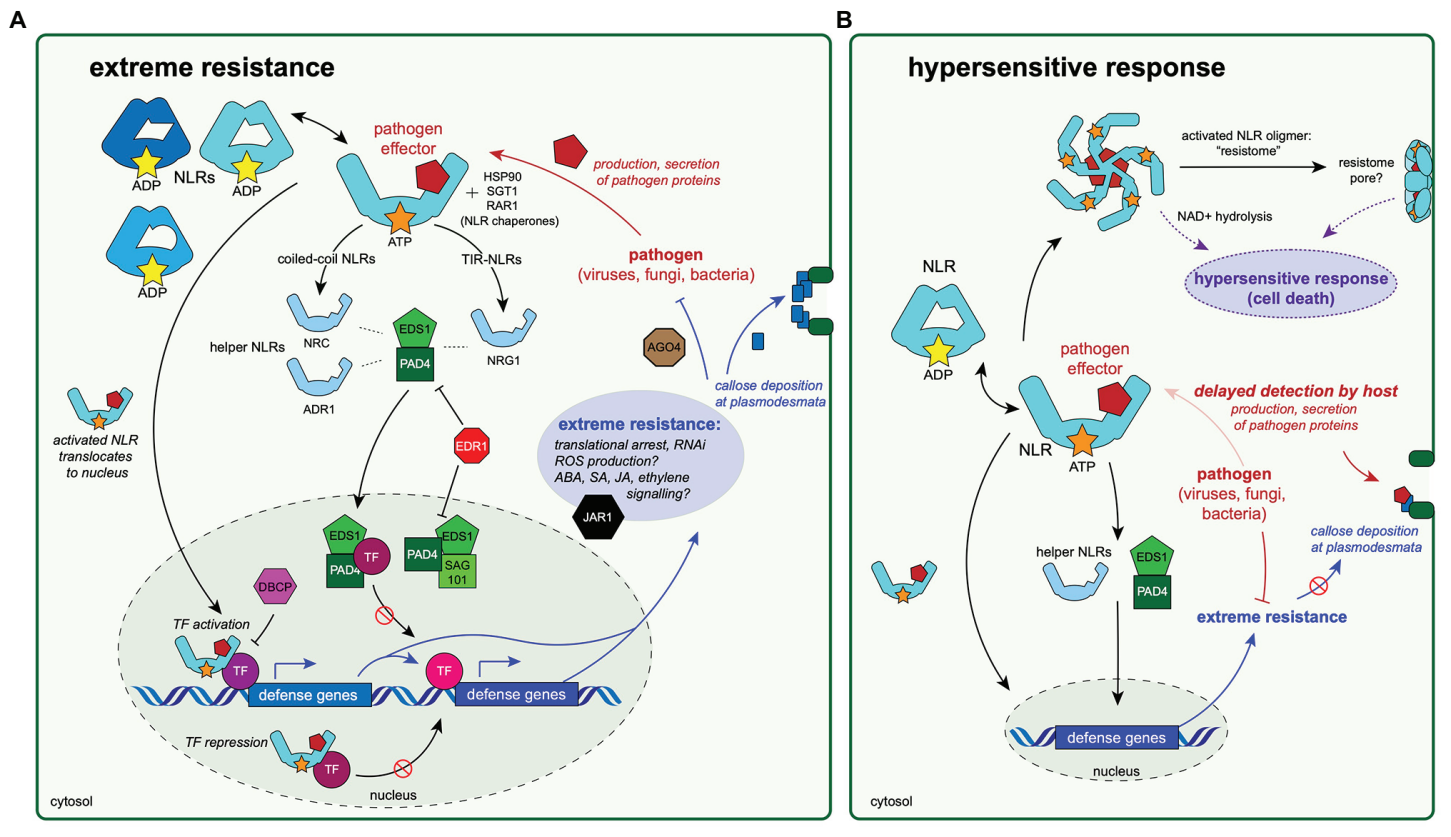
Diagrams of a hypothesized extreme resistance pathway and its possible relationship to the hypersensitive response. Experimental evidence to date indicates that the proteins highlighted in these diagrams are important for antiviral defenses mechanisms, including extreme resistance **(A)** and the hypersensitive response (HR; **B**). These two pathways may share specific protein components and/or activation mechanisms, and therefore, may be thought of as a continuum of antiviral response rather than two distinct pathways. The conserved mechanisms of the extreme resistance response remain largely unknown, although this review (and others; Kuenstler et al., 2016) have noted some unifying themes. **(A)** The extreme resistance response depends on NLR activation and recognition of a pathogen-produced effector protein. The activated NLR may then translocate to the nucleus, where it could form a complex with transcription factor(s), resulting in either the activation of defense responses by binding to DNA and promoting transcription of defense genes or by preventing DNA binding of transcription factors that repress defense gene expression. Immune signaling through EDS1/PAD4/SAG101 proteins and resulting complexes likely also plays a role in initiating immune responses, but the mechanisms are not understood. Activation of extreme resistance defense pathways results in translational arrest increased abscisic acid (ABA) signaling, which allows for callose deposition at plasmodesmata, increased expression of components of the RNA interference (RNAi) pathway, and likely other forms of hormonal signaling. **(B)** The relationship between extreme resistance and the HR is not well understood. The timing of the pathogen recognition event by the host may play a role in determining the phenotype of the resulting resistance response. Delayed recognition of the pathogen could allow for more production or secretion of pathogen-derived proteins, many of which are involved in disabling host immune responses, including preventing callose deposition, which is a key aspect of extreme resistance. Eventual recognition of many pathogen proteins could lead to the oligomerization of activated NLR complexes and the formation of resistome pore-like structures in the cell wall, which may play a role in HR (Adachi et al., 2019b).

Understanding plant immune responses is a critical component to developing disease-resistant crops and limiting losses due to pathogens. Herein, we review the current literature of mechanisms to extreme resistance to viruses in the economically important plant species, potato, and soybean. Although the mechanisms underlying extreme resistance are not well understood, there appear to be some possible unifying themes (as illustrated in Figure 2A):

1. **Nuclear translocation of an activated NLR post-pathogen recognition may be an important component of extreme resistance:** In particular, the NLR protein, Rx1, and its translocation to the nucleus following recognition of PVX coat protein. Further research on possible gene regulation by the nuclear-localized *Rx1-GLK1* complex after PVX detection could identify other genes involved in extreme resistance. Additional research into whether activated Ry_sto_, Rsv3, or proteins from individual genes isolated from the *Rsv1* locus translocate to the nucleus following pathogen recognition would provide insight as to if nuclear translocation of NLR proteins and gene regulation is a conserved aspect of the extreme resistance response.
2. **Key immune signaling components are shared between the HR and extreme resistance responses:** This review outlines the current literature regarding extreme resistance to particular strains of PVY and SMV as conferred by the *Ry_sto_* gene in potato and the *Rsv1* locus and *Rsv3* gene in soybean. Experiments that involved virus-induced gene silencing demonstrated that extreme resistance conferred by *Ry_sto_*, *Rx1*, *Rsv1*, and other NLR genes and extreme resistance loci rely on many of the same proteins as resistance provided by HR (e.g., helper NLRs, *PAD4*, *EDS1*, etc.). Furthermore, many of the NLR proteins that confer extreme resistance can also elicit HR. Future experiments that determine if *Rsv3*-conferred extreme resistance relies on similar mechanisms will be vital to elucidating if extreme resistance across plant species can be attributed to a defined series of molecular events, or if plants have evolved multiple strategies that result in an extreme resistance phenotype.
3. **The interplay between hormone signaling, callose deposition, and translational arrest could form the basis of the extreme resistance response:** Recent studies of extreme resistance to SMV and PVY indicates that virus recognition promotes increases in ABA signaling, which results in increased callose deposition at plasmodesmata, thus preventing viral spread from cell to cell. Increased ABA concentrations in soybeans was associated with increased expression of genes involved in RNAi, possibly resulting in targeted destruction of viral genomes (Figure 2A). It is noteworthy that virulent strains of PVY prevent callose deposition to promote infection. Further research into binding targets of extreme resistance-breaking virus strains will likely yield further insight into the mechanisms of extreme resistance. Likewise, virus-specific translational arrest appears to be an important component of preventing virus replication in extreme resistance conferred by *Rx1* (Bhattacharjee et al., 2009; Richard et al., 2021). The underlying mechanism(s) of translational arrest and the possible involvement of *Argonaute 4* are areas ripe for further research. Finally, genes involved in salicylic acid, ABA, and jasmonic acid signaling have all been implicated in the extreme resistance response. Determining the possible roles and interplay of these hormones during extreme resistance will provide for a better understanding of NLR-mediated virus defense.

There are many questions yet unanswered with regards to extreme resistance, particularly its relationship with HR. It appears that these seemingly distinct resistance phenotypes are connected and may represent ends of the plant immune response spectrum. Research indicates that the expression levels of both the NLR and the pathogen protein recognized by the NLR play roles in determining the phenotypic outcome of the interaction. The timing of immune activation could also be an important aspect in determining the resulting resistance phenotype. Delayed or inefficient recognition of pathogen infection may provide the pathogen with time to disrupt or disable early defense response (i.e., translational arrest, hormonal signaling, callose deposition, and RNAi), thus triggering HR (Figure 2B), but this idea remains largely untested.

Disease resistance is a growing focus of crop breeding programs around the world. Given the mounting challenges to global agriculture posed by a changing climate and a burgeoning human population, a greater understanding of plant defense responses to viruses will be valuable assets to breeders and growers alike.

## Author Contributions

BR, NZ, and MF developed the concept of this paper. BR wrote the original version of this manuscript, and BR, NZ, and MF reviewed and edited the manuscript. NZ and MF provided supervision and acquired funding for this work. All authors contributed to the article and approved the submitted version.

### Conflict of Interest

The authors declare that the research was conducted in the absence of any commercial or financial relationships that could be construed as a potential conflict of interest.
